# Immune skeletal dysplasia with neurodevelopmental abnormalities caused by a novel variant of *EXTL3* gene in a Chinese family

**DOI:** 10.1002/mgg3.2308

**Published:** 2023-11-27

**Authors:** Xinyuan Tian, Xiaoni Zhang, Qinghua Zhang, Xue Chen, Bingbo Zhou, Panpan Ma, Lei zheng, Shengju Hao, Junhe Ling, Chuan Zhang, Ling Hui

**Affiliations:** ^1^ Center for Medical Genetics Gansu Provincial Maternity and Child Care Hospital, Gansu Provincial Clinical Research Center for Birth Defects and Rare Diseases Lanzhou China; ^2^ School of Public Health Gansu University of Chinese Medicine Lanzhou China; ^3^ Center for Early Childhood Development Gansu Provincial Maternity and Child‐Care Hospital Lanzhou China

**Keywords:** *EXTL3* gene, immune skeletal dysplasia with neurodevelopmental abnormalities, immune system function abnormalities, neurodevelopmental deficits, skeletal abnormalities

## Abstract

**Background:**

Immune skeletal dysplasia with neurodevelopmental abnormalities (ISDNA) is an extremely rare, autosomal recessive genetic disorder characterized by various skeletal abnormalities, neurodevelopmental deficits, and abnormal immune system function. ISDNA is caused by variation in the exostosin‐like 3 (*EXTL3*) gene, located on chromosome 8p21.2, whose primary function is the biosynthesis of heparan sulfate (HS) skeleton structure. Only a few variations in the *EXTL3* gene have been discovered so far. In these years of development, many pathogenic variants in genetic diseases with genetic and phenotypic heterogeneity have been investigated using whole‐exome sequencing (WES) technology.

**Methods:**

In this research, a novel *EXTL3* variant was first detected in a patient using WES, which was validated from Sanger sequencing in this family. Family history and clinical information were then collected through comprehensive medical examinations and genetic counseling. In silico prediction was then utilized to confirm the pathogenicity of the variant.

**Results:**

A novel homozygous variant, NM_001440: c.2015G>A (p.Arg672Gln) in the *EXTL3* gene, was identified using WES, which has never been reported before. Sanger sequencing was performed to confirm that the variant segregated with the disease within the family.

**Conclusion:**

This research identified a novel pathogenic variant in the *EXTL3* gene responsible for ISDNA in a Chinese family. It showed the potential diagnostic role of WES in ISDNA, expanded the *EXTL3* gene variation spectrum, and demonstrated that the diagnosis of ISDNA using WES is feasible and effective. More comprehensive genetic counseling and precise prenatal diagnosis for the next pregnancy can also be provided to families with genetic disorders.

## Graphical abstract

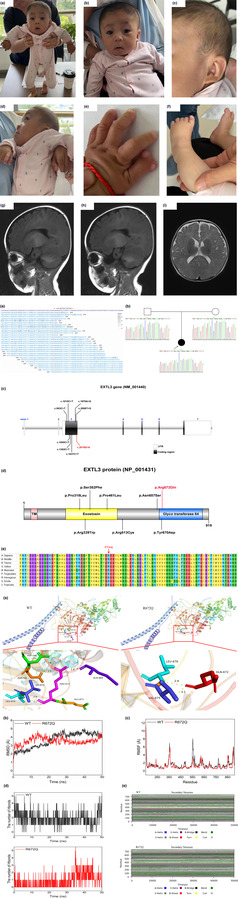

## 1 INTRODUCTION

Skeletal dysplasias are a class of monogenic disorders primarily involving the skeletal system and accounting for at least 436 diseases, with different patterns of inheritance and involvement. Subgroups of diseases under this regimentation involve immune dysfunction and skeletal dysplasia, such as spondyloenchondrodysplasia with immune dysregulation, Schimke immuno‐osseous dysplasia, cartilage‐hair hypoplasia and immune skeletal dysplasia with neurodevelopmental abnormalities (ISDNA, OMIM#617425). As a result of progressive immune dysfunction, infections and lung disorders are more likely to occur, causing a high mortality rate (Mäkitie et al., 2000).

ISDNA is a rare autosomal recessive genetic disorder caused by the variant in the exostosin‐like 3 (*EXTL3*) gene. It has been consistently linked to skeletal characteristics, including disproportionate skeletal dysplasia, odontoid hypoplasia, platyspondyly, epiphyseal and metaphyseal changes. Most of these characteristics are congenital and associated with immunodeficiencies and neurological features. The *EXTL3* gene (OMIM#605744) is located on chromosome 8p21.2, which consists of seven exons encoding the EXTL3 protein made up of 919 amino acids. This protein contains the GT64 and GT47 glycosyltransferase domains, the latter of which functions in both GlcNac transferase I and II activities (Awad et al., 2018). Its function is to biosynthesize the heparan sulfate (HS) backbone structure. It may also probably help in the signal transduction of REG protein (Yamada, 2020). The REG family of proteasome activators consists of three members: REG‐α, REG‐β and REG‐γ. The REG‐α and REG‐β exist mainly in the cytoplasm and are related to the immune response mediated by MHC‐1 in immune cells.

To date, only seven variants in the *EXTL3* gene have been identified in 18 cases of ISDNA: p.Pro318Leu, p.Arg339Trp, p.Ser352Phe, p.Pro461Leu, p.Arg513Cys, p.Asn657Ser, and p.Tyr670Asp. In this research, a Chinese patient was identified with ISDNA caused by a homozygous missense variant in the *EXTL3* gene, NM_001440: c.2015G>A (p.Arg672Gln), which was inherited from her parents.

## 2 MATERIALS AND METHODS

### 2.1 Editorial policies and ethical compliance

This research was approved by the Review Board of the Institutional Review Committee of Gansu Provincial Maternity and Child Health Hospital and conducted according to the tenets of the Declaration of Helsinki. Written informed consent to participate in this study was provided by the participants' legal guardian/next of kin. Written informed consent was obtained from the individual (s) and the minor (s)’ legal guardian of kin for the publication of any potentially identifiable images or data included in this article.

### 2.2 Whole‐exome sequencing

DNA was obtained from the peripheral blood of the patient and her parents using TIANGEN TIANamp Genomic DNA Kit, and the purity and concentration of DNA were determined using a NanoDrop 2000 nucleic acid quantifier (DNA concentration was controlled at 50–250 ng/μL). DNA was submitted to Chigene Co., Ltd. for trio whole‐exome sequencing (trio WES). The specific experimental methods are described in detail in another article published by the author (Tian et al., 2022).

### 2.3 Sanger sequencing

Primers were designed using online primer design software Primer 3 to cover the *EXTL3* gene (NM_001440) exon 3 and its flanking sequences (forward primer sequence: CAGGCAAGGCGGCTGGAACT; reverse primer sequence: ATGATGGGGACGCCAATGTCAGG). Further experimental information is detailed in another article published by the author (Tian et al., 2022).

### 2.4 In silico prediction

Illustrator for Biological Sequences v1.0 (IBS) was utilized to create the protein domain model diagram (Liu et al., 2015). The conservatism of the amino acid (R672) affected by c.2015G>A variant across species was illustrated using MEGA7. The 3D structural analysis of the wild‐type (WT) and mutant (MUT) domains of the EXTL3 protein was generated using PyMOL (Rigsby & Parker, 2016). The molecular dynamics (MD) simulation was conducted for both models. First, AmberTools were used to process the protein and generate topology and parameter files to prepare for the simulation. The ACPYPE6 program was then used to format these topology and coordinate files. Finally, molecular dynamics simulations were performed using GROMACS software (Lee et al., 2020; Sousa da Silva & Vranken, 2012).

## 3 RESULTS

### 3.1 Clinical information

**FIGURE 1:**
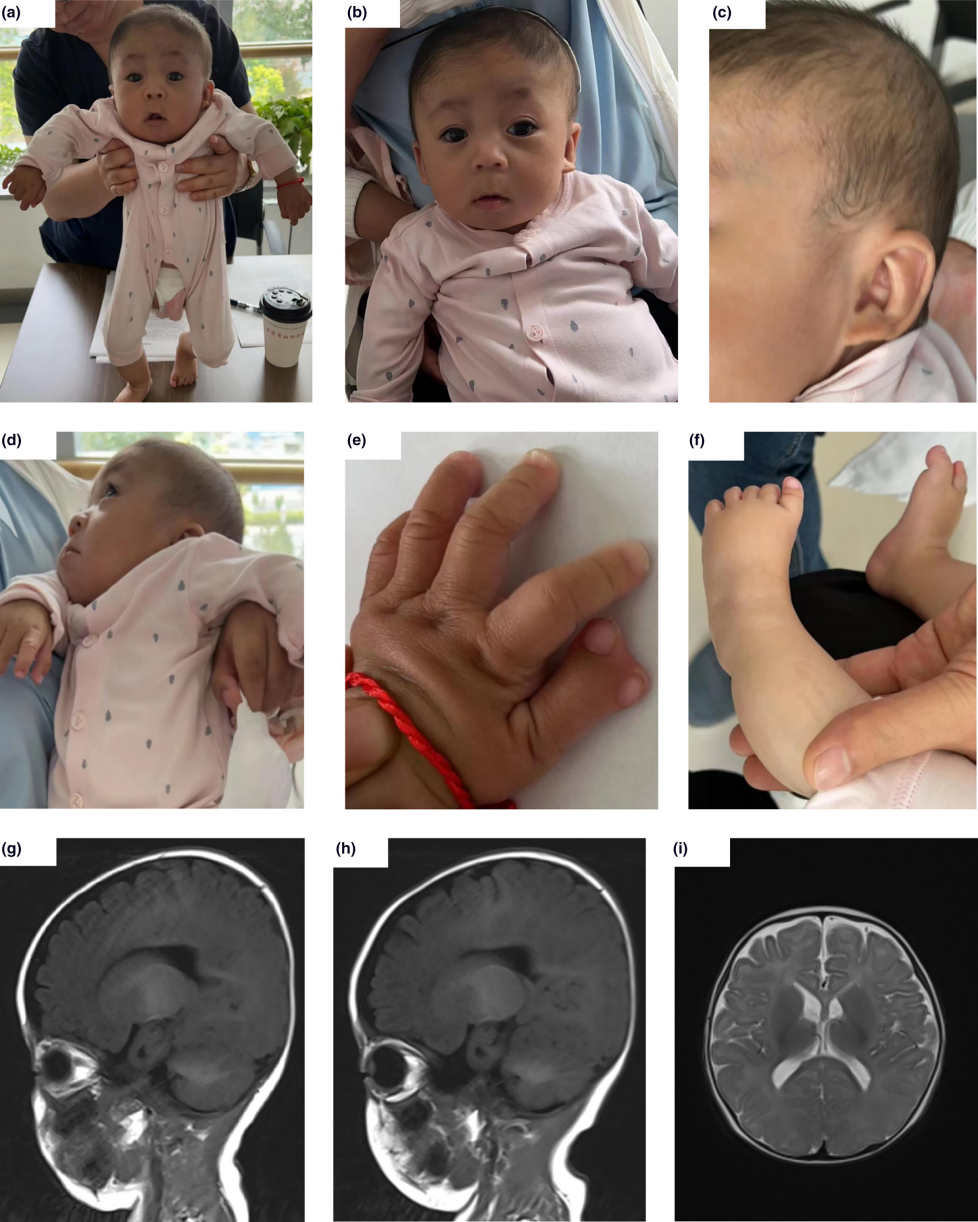
Clinical phenotypes of the patient. (a–f) Facial deformity: coarse face, micrognathia, high arched jaw, flat nose, long philtrum, and auricular deformity. (g–i) Features of cranial MRI (at the age of three months): hypoplasia of the corpus callosum.

A 3‐month‐old patient and her parents underwent genetic testing at the Medical Genetics Center of Gansu Maternal and Child Care Hospital. Her parents were healthy. The patient was born prematurely at 39 weeks of gestational age in our hospital. The prenatal fetal ultrasonography revealed dysplasia of right kidney and mild dilatation of the right lateral ventricle. The patient was born with neonatal hyperbilirubinemia, hypocalcemia, hypomagnesemia, cholestasis, and intracranial hemorrhage and had a poor postpartum response with shortness of breath. She was hospitalized for severe birth asphyxia, neonatal pneumonia, low birth weight, left thumb with multiple fingers and syndactyly. She had increased muscle tone in her extremities, was unable to raise her head when prone, and responded poorly to eye tracking and teasing. The bone mineral density of the patient was normal and no abnormality was found in bilateral hip joint ultrasound. Unfortunately, because of the refusal of the patient's family, we did not get an x‐ray picture about the patient's hand bones. Her right kidney was tiny (0.023*0.011 m) and deformed, with the right renal hilum facing anterolaterally. Her nasopharyngeal soft tissue collapsed, possibly laryngomalacia (moderate). Echocardiography revealed a patent ductus arteriosus with a central atrial septal defect in the patient. She had mild facial dysmorphic features of micrognathia, high arched jaw, flat nose, long philtrum and auricular deformity, as well as scattered rashes and skin lesions on her face and trunk (Figure 1a–f). Magnetic resonance imaging of the head revealed hypoplasia of the corpus callosum, an increased distance of the posterior horns of the bilateral lateral ventricles, a small volume of the inferior cerebellar vermis, and enlarged cisterna magna (Figure 1g–i). In the patient, the blood ammonia was elevated, and the hepatic transaminases were decreased. The CD3+ T cells decreased (43%), the CD3+CD4+ Th cells decreased (27%), the CD3+CD8+ Ts cells decreased (14%), and the CD3−CD19+ B cells increased (41%) were detected by the lymphocyte subgroup analysis. Through the examination of Th1/Th2 cytokines, we found that the levels of IL‐6, IL‐10 and TNF‐α were all increased (four times the normal value). The results of the antinuclear antibody spectrum were normal. Through the detection of the markers, the liver function of the patient was normal, and the results of FT3, FT4 and TSH indicated that there was no problem with thyroid function. Since birth, the patient has had repeated attacks of upper respiratory tract infection and pneumonia, but has not been cured so far. Based on the above results, there might be mild defects in the immunological function of the patients. Through follow‐up interviews, we learned that the patient had disproportionate symptoms of short stature (Table 1), and that she could not speak or walk at the 17 months age and was slow to respond. Our clinical suspicion included ISDNA.

**TABLE 1:** The evaluation of growth and development of the patient.

Age	Weight	Length	Head circumference	Standard value	BMI	Development assessment
0D	2.61 kg (−1.8SD)	0.47 m (−1.6SD)	0.31 m (−2.5SD)	−1.0SD	11.8 kg/m^2^ (−1.2SD)	Mild emaciation
3M	5.30 kg (−1.3SD)	0.55 m (−2.6SD)	0.39 m (−0.5SD)	1.0SD	16.5 kg/m^2^ (−0.2SD)	Normal
5M	5.90 kg (−2.0SD)	0.63 m (−1.0SD)	0.41 m (−0.5SD)	−1.3SD	14.8 kg/m^2^ (−2.0SD)	Moderate emaciation
6M	6.07 kg (−2.3SD)	0.64 m (0.6SD)	0.41 m (−1.2SD)	−1.8SD	14.8 kg/m^2^ (−2.0SD)	Moderate emaciation
7M	6.20 kg (−2.5SD)	0.66 m (−1.0SD)	0.44 m (0.4SD)	−2.4SD	14.2 kg/m^2^ (−2.5SD)	Moderate emaciation

Abbreviations: D, days old of age; F, female; M, male; M, months old of age; ND, not described; Y, years old of age.

### 3.2 Genetic analysis

**FIGURE 2:**
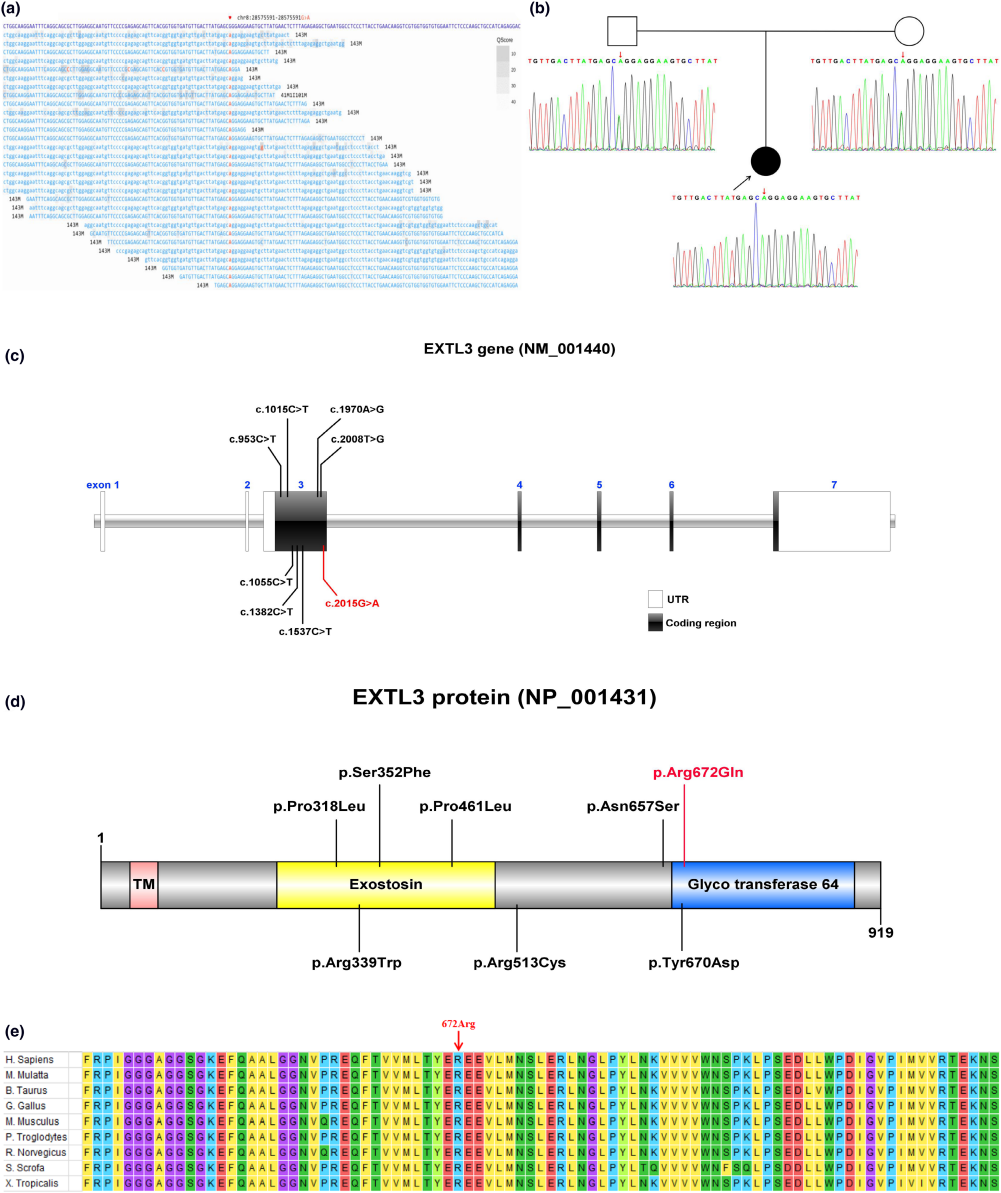
Genetic findings: (a) The WES data demonstrating the *EXTL3* c.2015G>A variant. (b) The pedigree and Sanger sequencing results of *EXTL3* c.2015 in the patient's family. A novel *EXTL3* c.2015G>A homozygous missense variant was detected in the patient with ISDNA, whose parents were both heterozygous for the variant at this locus. (c, d) The schematic diagram of *EXTL3* and the corresponding protein with all the reported variants till date. The variant in our research is shown in red. (e) The conservatism analysis of the amino acid Arg672 sites.

A homozygous missense variant c.2015G>A (p.Arg672Gln, p.R672Q) in the *EXTL3* gene was identified in the patient using WES. Furthermore, Sanger sequencing was used to confirm this identified variant. Both the patient's parents were found to carry this heterozygous variant (Figure 2a,b). The *EXTL3* gene (NM_001440) contains seven exons, of which exon 3 is the main one with coding functions. According to the literature and data collection, it was found that all variants occur in the coding region of exon 3, resulting in an aberrant structure and function of the EXTL3 protein (NP_001431). The variant site c.2015G>A was located in the coding region of exon 3 (Figure 2c). The transmembrane protein EXTL3 is composed three domains, transmembrane (TM) domain, exostosin (EXT) domain, and glycosyltransferase family 64 domain, which passes through the membrane once. Most of the reported ISDNA‐causing variants are concentrated in two predicted glycosyltransferase domains, particularly the conserved EXT domain. In this research, the variant site p.Arg672Gln was located in the glycosyl transferase family 64 domain (Figure 2d). The residue affected by this novel variant, R672, remains evolutionarily conserved across species (Figure 2e). It was predicted to prove “deleterious” to gene function through REVEL, Provean, and CADD. Accordingly, this variant was initially determined to be likely pathogenic and satisfied the evidence level PM1, PM2, PP3, and PM3‐Supporting (PM1: located in a mutational hot spot and/or critical and well‐established functional domain without benign variation; PM2: the frequency in all normal population database is <0.0005; PP3: multiple lines of computational evidence support a deleterious effect on the gene or gene product; PM3‐Supporting: this variant was homozygous).

### 3.3 The *EXTL3* R672Q variant impacting the protein stability and secondary structure

**FIGURE 3:**
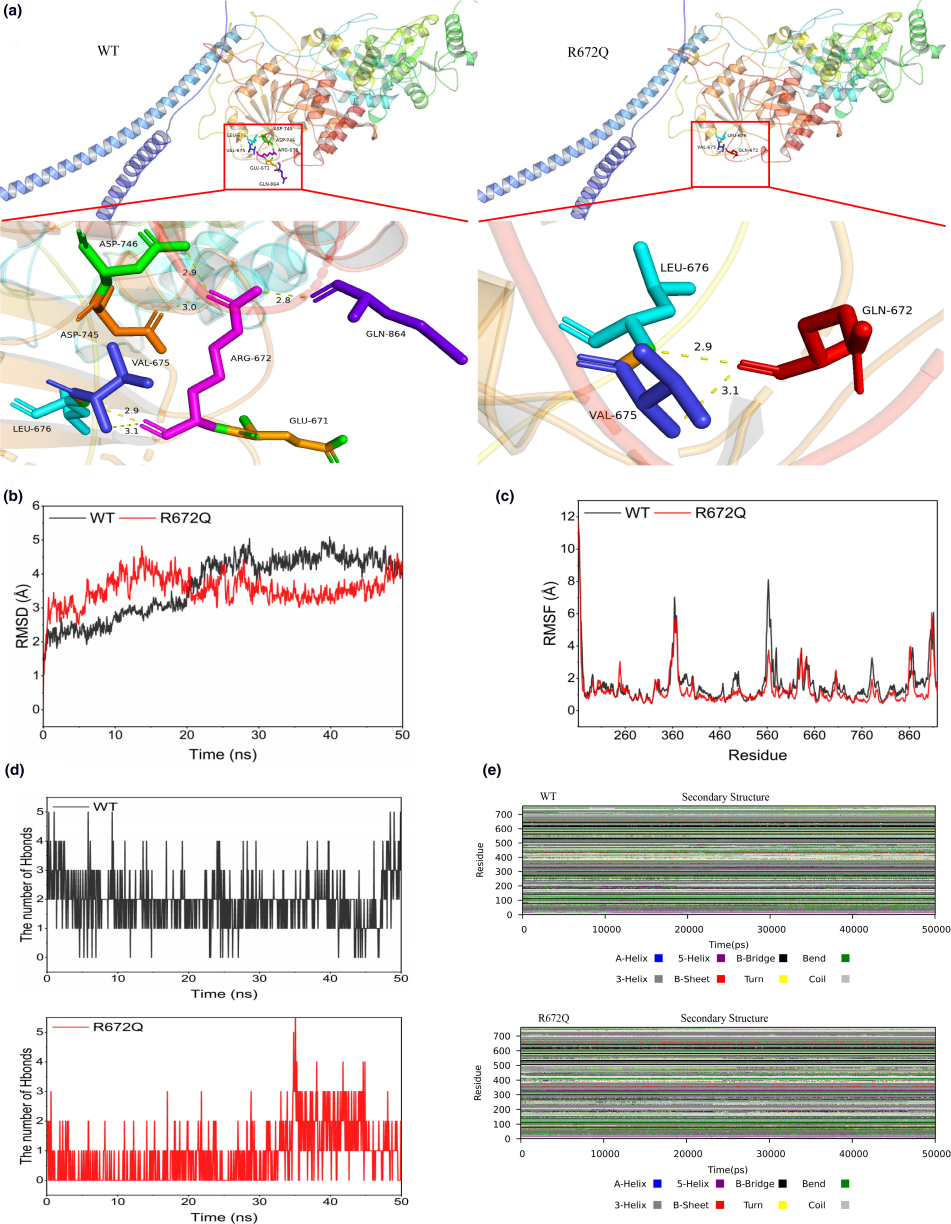
Structural and MD analyses of the EXTL3 p.Arg672Gln variant: (a) The structural analysis of the domain containing the WT and R672Q models. The dotted yellow lines represent the hydrogen bonds. (b) In MD, the RMSD refers to the structural deviation relative to the reference conformation at a particular time, showing the degree of change in the molecular structure. The trajectory of RMSD can be used to spot significant changes in protein structure relative to the initial state. (c) In MD, the RMSF refers to the structural changes of selected atoms relative to the reference conformation over a period of time, reflecting the degree of freedom of each atom in the molecule. It is a calculation of individual residue flexibility or fluctuations during a simulation. (d) The number of hydrogen bonds formed between the residue R672 or R672Q and the other residues, which are crucial to maintaining the structural stability of proteins and promoting interactions of molecules. (e) The secondary structural components of the corresponding region as a function of time. Using the trajectory files of the two models, we call the do_dssp command to calculate the change of protein secondary structure with time. We obtained the secondary structure information of each amino acid residue in each frame and the number of different secondary structure residues, and defined the color of each secondary structure over time. The corresponding secondary structure of the EXTL3 protein was also affected by the R672Q variation.

Structural analysis results revealed that the Arg672 interacted with Val675, Leu676, Asp745, Asp746, and Gln864 to form hydrogen bonds, which enhanced the stability of the peptide skeleton folding. However, the spatial conformation was significantly altered when Arg672 was substituted with Gln672 in mutant EXTL3, suggesting that Gln672 could not form hydrogen bonds with other amino acid residues in addition to Val675 and Leu676 (Figure 3a). Furthermore, the Arg is extremely basic and can easily form a salt bridge with extremely acidic Glu residues, so the *EXTL3* Arg672 variant results in defective salt bridge formed by Glu671. The change from a positively charged Arg to a neutral Gln may affect the regional environment, which could subsequently impair the permeation properties of EXTL3. Through molecular dynamics simulation, we could know the difference of protein structure stability between WT (R672) and MUT (R672Q) model. In these two models, we took the alpha‐C atom in the system as the selected atom and compared the conformational differences between them from different angles and aspects. Using the trend diagram of Root Mean Square Deviation (RMSD) and Root Mean Square Fluctuation (RMSF) trajectory, it was suggested that the protein conformation of R672Q model is not as stable as that of WT model (Figure 3b,c). By analyzing the number of hydrogen bonds, we found that the R672Q formed fewer hydrogen bonds with other amino acid residues than the WT model (Figure 3d). The secondary structure simulation diagram showed that with the change of time, the corresponding secondary structure of the EXTL3 protein was affected by the R672Q variation, which is different from WT model (Figure 3e). To sum up, compared with R672, the *EXTL3* mutant protein exhibited a reduced number of hydrogen bonds, weaker intermolecular forces, altered spatial conformation, and destroyed structural stability after the R672Q variation.

## 4 DISCUSSION AND CONCLUSION

The biallelic variations in the *EXTL3* gene can cause ISDNA, a rare genetic disorder that has been described in only seventeen cases worldwide. In this research, we report a female patient with ISDNA caused by an unreported homozygous variation c.2015G>A in the *EXTL3* gene. The *EXTL3* gene has seven exons, of which exon 3 occupies most of the coding regions and is responsible for encoding most of the EXTL3 protein, including functional domains. There is also a small portion of the UTR regions that does not encode proteins and only plays a role in the translation regulation function. The seven variant sites reported so far are all located in the coding region of exon 3, which is the hot spot of *EXTL3* gene variation. The c.2015G>A variant found in this study is also located in this region (Figure 2c).

The exostosin (EXT) proteins are glycosyltransferases in the Golgi apparatus that assembles HS chains on HSPGs. The *EXTL3* encodes a glycosyltransferase, primarily as an initiator of HS biosynthesis, synthesizes the backbone structure of HS, and plays a role in bone formation and bone remodeling. HS, a widely distributed sulfated polysaccharide on the surface of animal cells and in the extracellular matrix, affects the activity of several important biological molecules involved in cellular communication and signaling (Awad et al., 2018). Lack of EXTL3 reduces HS levels and causes embryonic lethality. EXTL3 has also been identified as a receptor molecule for ligands of regenerating islet‐derived (REG) proteins, which are crucial for keratinocyte proliferation and/or differentiation, tissue regeneration and immune defense in the gut, and neurite outgrowth in the central nervous system (Yamada, 2020). Experimental research in model organisms has confirmed the various roles of mutant EXTL3 proteins. These findings could explain the skeletal and immunological symptoms in patients with *EXTL3* gene variants (Volpi et al., 2017). EXTL3 can be used as an anticancer gene and is significantly associated with seven signaling pathways in prostate cancer. Therefore, EXTL3 is a potential biomarker for evaluating prognosis and immunotherapy in prostate cancer (Chang et al., 2022). The reported *EXTL3* variants were primarily distributed in the EXT domain, affecting the assembly of HS, intercellular communication and biological signal transduction, resulting in defects in the growth and development of the central nervous system and immune system, and abnormal skeletal development. The p.Arg672Gln variation found in this research was located in the glycosyltransferase family 64 domain of the EXTL3 protein, which may affect the assembly of the glycosyltransferase domain, causing functional defects of the EXTL3 protein and failure to synthesize HS smoothly (Figure 2d).

The p.Arg672 site is substantially conserved among all EXTL3s in different species (Figure 2e). It can form hydrogen bonds and salt bridges with nearby amino acid residues. Therefore, the p.Arg672Gln variant can result in hydrogen‐bonding defect and salt‐bridge breakage, leading to structural instability of EXTL3, restricted HS synthesis, and impaired bone formation and bone remodeling. It also affects the signal transduction of REG protein, resulting in abnormal development of the nervous system and immune system (Figure 3).

ISDNA is an autosomal recessive disorder characterized by several skeletal abnormalities and neurodevelopmental defects. Nervous system abnormalities include intellectual disability and motor retardation. The symptoms of hypotonia and seizures are observed in some patients. Skeletal abnormalities include disproportionate short stature, cervical deformity, epiphyseal and metaphyseal dysplasia, and occasional congenital anomaly of the anterior craniocerebral space with progressive microcephaly. Severe combined immunodeficiency and complete T‐cell deficiency can be observed in some patients. Furthermore, there are other clinical phenotypes, such as the rough face, full cheeks, wide‐set eyes, high nose, wide nose tip, sunken bridge, pectus excavatum, flat vertebrae, penetrating palms, exfoliative dermatitis, brachydactyly syndrome, liver cysts, small kidneys, anal atresia, eosinophilia, lymphopenia, recurrent infections, and hypogammaglobulinemia. The affected individuals experience severe morbidity due to the multisystem involvement. Recent studies have revealed that gene variants in ISDNA patients are all missense variants, and ISDNA has a high phenotypic heterogeneity (Table 2). Most patients had phenotypes, such as facial deformities, disproportionate short stature, neurodevelopmental deficits, musculoskeletal dysplasia, and limb abnormalities. But immune system deficiencies and abnormal liver and kidney development were not present in all patients. Some patients exhibit abnormal body wall manifestations, such as skin rash, skin lesions, and penetrating palms, while some exhibit abnormal cardiovascular system development and metabolic disorders. Although both are neurodevelopmental defects, there are phenotypic differences, such as intellectual disability, motor retardation, and seizures that may not occur simultaneously. Furthermore, the clinical manifestations of skeletal developmental malformations also have considerable differences. Additionally, the same locus variation can produce different degrees of phenotypic differences. Therefore, the occurrence of the ISDNA may also be linked to other genetic backgrounds, such as gene interactions, epigenetic changes, and environmental factors.

**TABLE 2:** Clinical phenotypes of ISDNA patients with the variation reported in the *EXTL3* gene.

Patient	Age	Variant	Gender	Craniofacial deformities	Disproportionately short stature	Nervous system abnormalities	Musculoskeletal system abnormalities	Limb abnormalities	Immune system abnormalities	Blood and hematopoietic tissue abnormalities	Digestive system abnormalities	Urogenital system abnormalities	Cardiovascular abnormalities	Body wall abnormalities	Eye abnormalities	Metabolic disorder	Study
1	5M	c.953C>T; p.Pro318Leu	F	Yes	Yes	Yes	Yes	Yes	Yes	Yes	Yes	No	nd	nd	No	No	(Guo et al., 2017)
2	8M	c.953C>T; p.Pro318Leu	F	Yes	Yes	Yes	Yes	Yes	No	No	Yes	No	Yes	nd	No	No	Guo et al. (2017)
3	15Y	c.953C>T; p.Pro318Leu	M	Yes	Yes	Yes	Yes	Yes	Yes	Yes	Yes	Yes	nd	nd	Yes	No	Akalın et al. (2021)
4	15M	c.953C>T; p.Pro318Leu	F	Yes	Yes	Yes	Yes	Yes	No	No	No	No	Yes	nd	Yes	No	Bajaj et al. (2022)
5	9M	c.1015C>T; p.Arg339Trp	F	Yes	Yes	Yes	Yes	Yes	No	Yes	nd	Yes	nd	Yes	nd	Yes	Volpi et al. (2017)
6	2M	c.1015C>T; p.Arg339Trp	F	Yes	Yes	Yes	Yes	Yes	No	Yes	nd	Yes	nd	Yes	nd	Yes	Volpi et al. (2017)
7	31Y	c.1055C>T; p.Ser352Phe	M	nd	nd	nd	Yes	Yes	nd	nd	Yes	nd	nd	nd	nd	nd	Pata et al. (2009)
8	2Y	c.1382C>T; p.Pro461Leu	F	Yes	Yes	Yes	Yes	Yes	No	Yes	nd	Yes	nd	Yes	nd	Yes	Volpi et al. (2017)
9	12Y	c.1382C>T; p.Pro461Leu	F	Yes	nd	Yes	Yes	Yes	Yes	Yes	nd	No	Yes	nd	nd	nd	Oud et al. (2017)
10	12Y	c.1382C>T; p.Pro461Leu	F	Yes	Yes	Yes	Yes	Yes	nd	nd	Yes	nd	Yes	Yes	nd	No	Oud et al. (2017)
11	22Y	c.1382C>T; p.Pro461Leu	M	Yes	nd	Yes	Yes	Yes	nd	nd	nd	nd	nd	nd	nd	No	Oud et al. (2017)
12	38Y	c.1382C>T; p.Pro461Leu	F	nd	nd	Yes	Yes	Yes	nd	nd	nd	nd	nd	nd	nd	No	Oud et al. (2017)
13	49D	c.1537C>T; p.Arg513Cys	F	nd	Yes	Yes	Yes	Yes	Yes	Yes	Yes	Yes	nd	nd	nd	nd	Oud et al. (2017)
14	6M	c.1970A>G; p.Asn657Ser	M	nd	Yes	Yes	Yes	Yes	Yes	Yes	Yes	Yes	nd	Yes	nd	nd	Oud et al. (2017)
15	10M	c.1970A>G; p.Asn657Ser	M	nd	nd	Yes	Yes	Yes	Yes	Yes	Yes	Yes	nd	Yes	nd	nd	Oud et al. (2017)
16	8Y	c.2008T>G; p.Tyr670Asp	M	Yes	Yes	Yes	Yes	Yes	Yes	Yes	Yes	No	No	Yes	No	No	Oud et al. (2017)
17	2Y	c.2008T>G; p.Tyr670Asp	F	Yes	Yes	Yes	Yes	Yes	Yes	Yes	No	Yes	Yes	Yes	nd	nd	Oud et al. (2017)
18	3M	c.2015G>A; p.Arg672Gln	F	Yes	Yes	Yes	Yes	Yes	Yes	Yes	No	Yes	Yes	Yes	Yes	Yes	This study

Abbreviations: D, days old of age; F, female; M, male; M, months old of age; nd, not described; Y, years old of age.

The STRING database showed interactions between EXTL3 and EXTL1, EXTL2, NDST1, HS3ST5, TRAP1, UXS1, SLC35B2, HS2ST1, GLCE and XBP1, which included both direct physical interactions and indirect functional associations. Furthermore, REG3A is known to bind and interact with EXTL3 to activate the downstream PI3K‐AKT signaling pathway, which subsequently regulates keratinocyte proliferation and differentiation after skin injury (Lai et al., 2012). The function of REG3A may be negatively affected by defects in the structure and function of EXTL3, resulting in phenotypes such as dermatitis and skin lesions. Hence, in the ISDNA caused by EXTL3, some patients also showed abnormal body wall, in addition to damage phenotypes in the main immune, musculoskeletal, and nervous system. It can be seen that the specific phenotypic abnormality and severity need to be comprehensively judged in combination with the interaction of other genes. Our patients had abnormalities in neurodevelopment, musculoskeletal development, immune system function, decreased T lymphocytes, development of liver, kidney, and cardiovascular, minor facial deformities, and body wall, expanding the phenotypic spectrum of *EXTL3* variants. In addition, we found other gene variants in the patient (Table S1), the *EP300* gene (OMIM#605894) c.2293C>T (p.Leu765Phe) and *FOXP3* gene (OMIM#300292) c.376C>A (p.Pro126Thr), both inherited from the mother. Variations in the *EP300* gene can cause syndactyly, and variants in the *FOXP3* gene can cause seizures and immune system abnormalities. Therefore, variations in these two genes may aggravate the patient's clinical phenotype.

Overall, this research reported the 18th ISDNA patient worldwide with a novel *EXTL3* gene variation, expanding the *EXTL3* gene variant spectrum and demonstrating that the diagnosis of ISDNA using WES is feasible and effective, and can provide precise genetic counseling and prenatal diagnosis for families with genetic diseases. The patient had mild facial deformities, skeletal dysplasia, neurodevelopmental disorders, and immunodeficiency, consistent with the symptoms of previously reported cases. Although all affected individuals showed similar neurological, skeletal, and immune system developmental abnormalities, variation in the context of genetic and/or environmental modifiers might have differential effects on the function of the mutant EXTL3 protein, as the patients' phenotypes were significantly differentiated. Therefore, it is difficult to determine the severity of the disease based on the location of the variant alone. Currently, there is no definitive cure for this disease other than supportive care and surgical intervention. It will be possible to understand the pathophysiology of this rare disease by reporting further cases and studies on mutant EXTL3 proteins.

## AUTHOR CONTRIBUTIONS

Chuan Zhang, and Ling Hui designed the research. Bingbo Zhou, and Junhe Ling used the software for bioinformatics evaluations. Qinghua Zhang, and Xue Chen performed the genetic analysis. Xinyuan Tian drafted the original manuscript. Xiaoni Zhang reviewed and improved the manuscript. Lei Zheng, Shengju Hao, and Panpan Ma collected the clinical data.

## FUNDING INFORMATION

This work was supported by the Science and Technology Program of Gansu Province (Grant No. 21JR7RA680), the National Key Research and Development Program of China (Grant No. 2016YFC1000307), the Lanzhou Science and Technology Plan Project (Grant No. 2021‐1‐182), the National Population and Reproductive Health Science Data Center (Grant No. 2005DKA32408), the Lanzhou Science and Technology Plan Project (Grant No. 2022‐5‐81), and the Lanzhou Talent Innovation and Entrepreneurship Project (Grant No. 2018‐RC‐95).

## CONFLICT OF INTEREST STATEMENT

There are no competing interests for any author.

## ETHICS STATEMENT

This research was approved by the Review Board of the Institutional Review Committee of Gansu Provincial Maternity and Child Health Hospital and conducted according to the tenets of the Declaration of Helsinki. Written informed consent to participate in this study was provided by the participants’ legal guardian/next of kin. Written informed consent was obtained from the individual (s) and the minor (s)’ legal guardian of kin for the publication of any potentially identifiable images or data included in this article.

## Supporting information


Table S1

## Data Availability

The original contributions presented in the study are included in the article/Supplementary Material; further inquiries can be directed to the corresponding author.
